# Psychological outcomes of smoking cessation supported by telemedicine in real-world clinical practice: A prospective longitudinal cohort study

**DOI:** 10.18332/tid/219641

**Published:** 2026-05-22

**Authors:** Serap Duru, Beyza Kerem, Ayşe Demirkaya, Gülistan Aydın

**Affiliations:** 1 University of Health Sciences, Etlik City Hospital, Ankara, Turkey

**Keywords:** depression, anxiety, cytisine, smoking cessation, telemedicine

## Abstract

**INTRODUCTION:**

Concerns about possible adverse psychological consequences of smoking cessation – particularly related to depression and anxiety – may act as barriers to quit attempts, reduce adherence to treatment, and negatively affect long-term abstinence. Our objective is to assess changes in depression and anxiety levels over a six-month follow-up period among adults attending a smoking cessation clinic under routine clinical practice conditions and to describe the role of scheduled telemedicine-based follow-up in supporting cessation outcomes.

**METHODS:**

This prospective observational cohort study enrolled 500 adult smokers who presented to a specialized smoking cessation outpatient clinic. Symptoms of depression and anxiety were evaluated using the Beck Depression Inventory (BDI) and Beck Anxiety Inventory (BAI) at baseline and at six months. In addition to face-to-face clinic visits, patients receiving medical treatment were followed through structured telemedicine contacts conducted at two-week intervals throughout the follow-up period.

**RESULTS:**

At six months, 180 participants (36.0%) achieved smoking cessation, while 320 (64.0%) continued smoking. Baseline demographic and clinical characteristics were similar between groups. In the overall cohort, median BDI scores declined from 12 (interquartile range, IQR: 8–17) at baseline to 8 (IQR: 5–12) at follow-up, and median BAI scores decreased from 11 (IQR: 7–16) to 7 (IQR: 4–11). Improvements in both depression and anxiety scores were observed in quitters and non-quitters, with greater reductions among individuals who successfully quit smoking. Cytisine was the most frequently prescribed pharmacological treatment (70.6%), and behavioral counseling was provided to all participants. Treatment-related adverse effects were reported by 21.8% of participants, and no serious adverse events occurred.

**CONCLUSIONS:**

Under real-world clinical conditions, smoking cessation was accompanied by meaningful reductions in depression and anxiety over a six-month period. Regular telemedicine-based follow-up conducted at two-week intervals appeared to facilitate treatment continuity and may enhance cessation outcomes.

## INTRODUCTION

Tobacco use continues to impose a substantial global health burden and remains one of the most preventable causes of premature morbidity and mortality worldwide. Contemporary Global Burden of Disease (GBD) analyses consistently rank cigarette smoking among the leading modifiable risk factors contributing to years of life lost and disability-adjusted life years across diverse regions and populations^1,2^. Moreover, recent GBD-based modeling studies suggest that even relatively modest reductions in smoking prevalence may translate into meaningful gains in population-level life expectancy, underscoring the urgent need for effective, scalable, and sustainable smoking cessation strategies integrated into routine healthcare systems^3,4^.

Nicotine dependence is now widely considered as a chronic, relapsing condition driven by complex neurobiological adaptations rather than a simple habit maintained solely by reward-seeking behavior. Current models highlight the involvement of multiple neural circuits beyond mesolimbic dopaminergic pathways, including habenula–interpeduncular signaling, glutamatergic modulation, and stress-responsive systems that collectively influence withdrawal severity, affective regulation, and relapse vulnerability^5-7^. During smoking cessation, the emergence of withdrawal-related symptoms – such as anxiety, dysphoria, irritability, sleep disturbances, and impaired concentration – often peaks in the early phase and may compromise cessation success, particularly among individuals with heightened psychological susceptibility^8,9^.

Accumulating evidence suggests a close and potentially bidirectional relationship between cigarette smoking and mental health outcomes. Large-scale longitudinal cohorts and Mendelian randomization studies indicate that smoking may increase the risk of depressive symptoms and certain psychiatric disorders, while pre-existing psychological distress may also predispose individuals to smoking initiation and persistence^10-12^. Importantly, unsuccessful quit attempts have been associated with transient worsening of depression and anxiety, highlighting the need to better characterize psychological trajectories during cessation efforts rather than focusing solely on abstinence outcomes^13^. In this context, the systematic assessment of psychological symptoms using validated instruments may provide clinically relevant insights into treatment adherence, relapse risk, and patient-centered outcomes^14^.

Evidence-based smoking cessation care relies on the integration of pharmacological therapy and behavioral support. The World Health Organization’s 2024 clinical treatment guideline for tobacco cessation endorses several effective pharmacotherapies, including nicotine replacement therapy, varenicline, bupropion, and cytisine, while acknowledging variability in accessibility and implementation across healthcare settings^15^. Cytisine, a plant-derived partial agonist of α4β2 nicotinic acetylcholine receptors, has gained renewed attention due to its demonstrated efficacy, favorable safety profile, and low cost, leading to increasing adoption in real-world clinical practice. Recent systematic reviews and meta-analyses have confirmed that cytisine is more effective than placebo and exhibits cessation outcomes comparable to established pharmacotherapies^16-18^. However, most clinical trials have prioritized abstinence endpoints, whereas data on psychological symptom trajectories – particularly changes in depression and anxiety during cytisine-supported cessation – remain limited. Moreover, in routine clinical settings, cytisine is frequently combined with behavioral counseling and structured follow-up strategies, making it difficult to disentangle the psychological effects of individual treatment components^19^.

The first six months following a quit attempt represent a particularly vulnerable period marked by elevated relapse risk, during which psychological factors play a decisive role in treatment continuity and long-term abstinence^8,13^. In recent years, telemedicine-based follow-up has emerged as a promising adjunct to conventional cessation services, offering opportunities for regular contact, timely reinforcement, and early identification of psychological distress. Despite growing interest, real-world data evaluating psychological outcomes in smoking cessation programs incorporating structured telemedicine follow-up remain scarce^20,21^.

Against this background, the present study aimed to evaluate changes in depression and anxiety levels over a six-month follow-up period among adults attending a smoking cessation clinic under routine clinical practice conditions. In addition, the study sought to describe cessation outcomes within a comprehensive real-world care model integrating pharmacological treatment, behavioral counseling, and scheduled telemedicine-based follow-up at two-week intervals. Rather than examining the isolated efficacy of a single pharmacological agent, this study focuses on the early psychological outcomes of smoking cessation as it occurs in everyday clinical practice and explores their association with cessation success.

## METHODS

### Study design, setting, and participants

This study was designed as a prospective, observational cohort study conducted at University of Health Sciences, Etlik City Hospital, Turkey. Owing to the observational nature of the study, no randomization or experimental intervention was performed. All treatment decisions were made in accordance with routine clinical practice and patient preferences.

Adult smokers aged ≥18 years who consecutively presented to the smoking cessation clinic during the study period were screened for eligibility. Inclusion criteria were: 1) current cigarette smoking, 2) willingness to initiate a smoking cessation attempt, and 3) adequate cognitive capacity to complete psychometric assessments. Exclusion criteria included the presence of severe psychiatric disorders (such as psychotic disorders or bipolar disorder), significant cognitive impairment, active malignancy, pregnancy or breastfeeding, and any condition that could interfere with study participation or follow-up. A total of 500 adult participants meeting the eligibility criteria were enrolled. All participants provided written informed consent prior to inclusion in the study.

### Smoking cessation interventions and follow-up

Smoking cessation interventions were delivered according to standard clinical practice. Pharmacological and behavioral treatment strategies were determined by the treating physician in collaboration with the patient, based on clinical assessment and patient preference. The majority of participants received cytisine-based pharmacotherapy, while others were treated with nicotine replacement therapy (NRT), behavioral counseling alone, or combination approaches. Behavioral counseling was provided to all participants as part of routine care.

In addition to face-to-face visits, follow-up of patients receiving pharmacological treatment was continued via telemedicine-based consultations at two-week intervals throughout the treatment period. These remote follow-up visits were used to monitor treatment adherence, address adverse effects, reinforce behavioral counseling, and provide motivational support. Telemedicine-assisted follow-up was integrated as a supportive component of routine care and aimed to enhance continuity of treatment.

Given the real-world design of the study, treatment modalities were not standardized, and no attempt was made to isolate the independent effects of individual therapeutic interventions.

### Data collection

Baseline data were collected during face-to-face interviews using standardized data collection forms. Sociodemographic variables included age, sex, marital status, and education level. Clinical data comprised anthropometric measurements (height, weight, body mass index), smoking-related characteristics (daily cigarette consumption, pack-years, duration of smoking, previous quit attempts, and passive smoke exposure), and the presence of chronic comorbid medical conditions.

Participants were followed for six months after initiation of the smoking cessation attempt. Smoking status at follow-up was determined primarily by self-report and supported by structured clinical interviews. In cases of suspected inconsistency, additional clarification was obtained during follow-up contacts.

### Psychological assessment

Depression and anxiety levels were assessed using the Beck Depression Inventory (BDI) and the Beck Anxiety Inventory (BAI), respectively. Both instruments are widely validated self-report scales commonly used in smoking cessation research. Assessments were performed at baseline (prior to treatment initiation) and at the follow-up visit at six months. Higher scores indicate greater severity of depressive or anxiety symptoms.

### Outcomes

The primary outcome of the study was smoking cessation status at six months, classifying participants as quitters or non-quitters. Secondary outcomes included changes in BDI and BAI scores over the six-month follow-up period, treatment adherence, and the occurrence of treatment-related adverse effects.

### Statistical analysis

Statistical analyses were performed using IBM SPSS Statistics version 26.0 (IBM Corp., Armonk, NY, USA). The distribution of continuous variables was assessed using the Kolmogorov–Smirnov test. Continuous variables were expressed as mean ± standard deviation (SD) for normally distributed data and as median with interquartile range (IQR) for non-normally distributed data. Categorical variables are presented as frequencies and percentages.

Within-group changes in BDI and BAI scores between baseline and six months were evaluated using the Wilcoxon signed-rank test. Between-group comparisons of changes in psychometric scores (Δ change) between quitters and non-quitters were performed using the Mann–Whitney U test. Categorical variables were compared using the chi-squared test. A two-sided p<0.05 was considered statistically significant.

## RESULTS

### Study population

A total of 500 adult smokers were included in the analysis, and all participants completed the 6-month follow-up. At six months, 180 participants (36.0%) reported complete smoking cessation, while 320 participants (64.0%) continued to smoke. The mean age of the study population was 42.9 ± 10.8 years. Overall, 272 participants (54.4%) were male and 228 (45.6%) were female.

Baseline demographic and clinical characteristics of the total cohort and according to smoking cessation status are summarized in Table 1. No substantial differences were observed between quitters and non-quitters with respect to age, sex distribution, marital status, education level, body mass index, or the prevalence of chronic comorbid conditions.

**Table 1:** Demographic and clinical characteristics of participants (N=500)

*Variables*	*Total* *%*	*Quitters* *%*	*Non-Quitters* *%*
**Total**, n	500	180	320
**Age** (years), mean ± SD	42.9 ± 10.8	43.0 ± 11.0	42.8 ± 10.7
**Sex** (males/females)	272/228	97/83	175/145
**Marital status** (married)	62.0	64.0	61.2
**Education** (≥ high school)	58.7	60.1	57.9
**BMI** (kg/m²), mean ± SD	26.4 ± 3.9	26.2 ± 3.8	26.6 ± 4.0
**Daily cigarettes**, mean ± SD	26.1 ± 9.4	24.8 ± 8.9	27.0 ± 9.6
**Pack-years**, mean ± SD	22.8 ± 11.6	21.5 ± 11.2	23.5 ± 11.8
**Passive exposure**	41.3	39.8	42.2
**Previous quit attempt**	58.3	59.4	57.2
**Cytisine use**	70.6	71.1	70.3
**NRT use**	30.1	28.9	31.0
**Behavioral counselling**	100	100	100
**Treatment adherence**	82.3	85.2	80.6
**Reported side effects**	21.6	22.8	21.0

BMI: body mass index. NRT: nicotine replacement therapy.

### Smoking characteristics

At baseline, the mean number of cigarettes smoked per day in the total cohort was 26.1 ± 9.4, and the mean cumulative smoking exposure was 22.8 ± 11.6 pack-years. Mean daily cigarette consumption was 24.6 ± 8.8 among quitters and 27.1 ± 9.7 among non-quitters. Mean cumulative smoking exposure was 21.3 ± 11.1 pack-years in quitters and 23.9 ± 11.9 pack-years in non-quitters.

Previous smoking cessation attempts were reported by 290 participants (58.0%). Passive smoke exposure was reported by 207 participants (41.3%). Baseline smoking-related characteristics are detailed in Table 1.

### Smoking cessation treatments and follow-up

Pharmacological treatment with cytosine was administered to 353 participants (70.6%), while 150 participants (30.0%) received nicotine replacement therapy. Behavioral counseling was provided to all participants as part of routine clinical care. Combination therapy with cytosine and nicotine replacement products was used in 175 participants (35.0%). The distribution of smoking cessation treatment modalities is presented in Table 2.

**Table 2:** Distribution of smoking cessation treatment modalities (N=500)

*Treatment modality*	*n*	*%*
Cytisine	353	70.6
Nicotine replacement therapy (NRT)	150	30.0
Behavioral counseling	500	100
Combination therapy (Cytisine + NRT)	175	35.0

Treatment modalities were not mutually exclusive, and some participants received combination therapy.

In addition to standard outpatient follow-up visits, patients receiving medical treatment were monitored through structured telemedicine follow-up. Participants were contacted at scheduled intervals every 15 days via telephone or remote consultation platforms. These follow-up contacts were conducted by trained healthcare professionals, focused on treatment adherence, symptom assessment, smoking status, and early identification of adverse effects, and were performed in a planned and appointment-based manner throughout the follow-up period. Complete treatment adherence was reported by 412 participants (82.3%).

### Psychological measures

At baseline, the median Beck Depression Inventory (BDI) score in the total cohort was 12 (IQR: 8–17), and the median Beck Anxiety Inventory (BAI) score was 11 (IQR: 7–16). At the follow-up at month 6, median BDI and BAI scores in the total cohort were 8 (IQR: 5–12) and 7 (IQR: 4–11), respectively. Among participants who quit smoking, the median BDI score decreased from 13 (IQR: 9–18) at baseline to 7 (IQR: 4–11) at month 6. The median BAI score in this group changed from 12 (IQR: 8–17) at baseline to 6 (IQR: 3–10) at month 6. Among participants who continued smoking, the median baseline BDI score was 11 (IQR: 7–16), and the median score at month 6 was 9 (IQR: 6–14). The median BAI score in this group was 10 (IQR: 6–15) at baseline and 8 (IQR: 5–12) at month 6. Between-group comparisons of changes in BDI and BAI scores over time are summarized in Table 3.

**Table 3:** Changes in depression and anxiety scores at baseline and 6 months by smoking status (N=500)

*Measurement*	*Time*	*Total* *Median (IQR)*	*Quitters* *Median (IQR)*	*Non-Quitters* *Median (IQR)*	p*
**Total**, n		500	180	320	
**BDI**	Baseline	12 (8–17)	13 (9–18)	11 (7–16)	-
**BDI**	Month 6	8 (5–12)	7 (4–11)	9 (6–14)	-
**BDI**	Δ Change	-4	-6	-2	<0.001
**BAI**	Baseline	11 (7–16)	12 (8–17)	10 (6–15)	-
**BAI**	Month 6	7 (4–11)	6 (3–10)	8 (5–12)	-
**BAI**	Δ Change	-4	-6	-2	<0.001

Δ Change represents the difference between baseline and month 6 scores. BDI: Beck Depression Inventory. BAI: Beck Anxiety Inventory. IQR: interquartile range.

* p-values refer to between-group comparisons of Δ Change (Mann–Whitney U test).

### Adverse events

Treatment-related adverse effects were reported by 109 participants (21.8%). The most frequently reported adverse effects were dry mouth (17.2%), mild nausea (15.0%), headache (12.4%), and sleep disturbances (9.4%). No serious adverse events were reported during the follow-up period. The frequency and distribution of reported adverse effects are presented in Table 4. Among participants who received cytosine-based pharmacotherapy (n=353), 110 individuals achieved self-reported smoking cessation at the follow-up at month 6, corresponding to a cessation rate of approximately 31%.

**Table 4:** Reported treatment-related adverse effects during follow-up (N=500)

*Adverse effect*	*n*	*%*
Dry mouth	86	17.2
Mild nausea	75	15.0
Headache	62	12.4
Sleep disturbances	47	9.4
No adverse effects reported	391	78.2

Participants could report more than one adverse effect.

## DISCUSSION

In this prospective real-world observational study, changes in depression and anxiety levels during the first six months of a smoking cessation attempt were evaluated among adult smokers attending a specialized cessation clinic. The observed reductions in depressive and anxiety symptoms across the follow-up period are consistent with accumulating evidence suggesting that smoking cessation is associated with improvements in mental health outcomes rather than psychological deterioration^22,23^. These findings are particularly relevant in real-life clinical settings, where concerns about potential worsening of mood symptoms may discourage quit attempts or compromise treatment adherence.

The association between smoking behavior and mental health is increasingly recognized as bidirectional. Large-scale longitudinal cohort studies and Mendelian randomization analyses indicate that cigarette smoking may contribute to the development and persistence of depressive symptoms, while baseline psychological distress may also increase susceptibility to smoking initiation and maintenance^12,24^. This evolving evidence challenges earlier beliefs that smoking functions as an effective coping strategy for emotional regulation and instead supports the hypothesis that chronic nicotine exposure may exacerbate affective dysregulation through neurobiological and behavioral mechanisms.

In the present cohort, reductions in Beck Depression Inventory and Beck Anxiety Inventory scores were observed among both participants who achieved smoking cessation and those who continued smoking, with greater improvements noted among quitters. Similar patterns have been reported in previous observational studies, where engagement in cessation-related behavioral change itself was associated with short-term psychological benefits, even in the absence of sustained abstinence^25^. Nevertheless, the more pronounced symptom reductions observed among quitters align with contemporary neurobiological models proposing that prolonged abstinence disrupts cycles of nicotine-induced mood fluctuation, withdrawal-related distress, and stress reactivity^26^.

Pharmacological and behavioral interventions were delivered according to routine clinical practice, with cytisine used in the majority of participants. Cytisine, a partial agonist of α4β2 nicotinic acetylcholine receptors, has demonstrated efficacy for smoking cessation in randomized controlled trials and meta-analyses^16,17,27^. In the present study, approximately one-third of participants receiving cytisine achieved smoking cessation, which is consistent with real-world effectiveness data reported in previous observational cohorts. However, as treatment modalities were not standardized and combination therapies were frequently used, the findings should not be interpreted as reflecting the isolated efficacy of cytisine but rather the outcomes of comprehensive cessation care.

An important and distinctive feature of this study is the integration of structured telemedicine-based follow-up into routine smoking cessation care. Following initiation of pharmacological treatment, participants were monitored via scheduled telemedicine contacts at two-week intervals, in addition to in-person clinic visits. This approach allowed for regular symptom assessment, treatment reinforcement, early identification of adverse effects, and timely behavioral support. Growing evidence suggests that telemedicine and digital health interventions can enhance treatment adherence, improve quit rates, and provide scalable solutions for tobacco dependence management, particularly during the early high-risk period following quit attempts^20,28^.

The first six months after smoking cessation initiation represent a critical phase characterized by heightened vulnerability to relapse. Psychological factors, including fluctuations in mood and anxiety, play a central role in treatment adherence and long-term abstinence during this period. By combining pharmacological therapy with frequent telemedicine-based follow-up, the present study reflects an emerging care model that addresses both biological and psychological dimensions of nicotine dependence. These findings support the growing consensus that telemedicine-based monitoring should be considered an integral component of modern smoking cessation services rather than a supplementary tool.

Overall, this study contributes real-world evidence demonstrating that smoking cessation, when supported by combined pharmacological treatment, behavioral counseling, and structured telemedicine follow-up, is associated with meaningful improvements in psychological well-being. Incorporating routine mental health assessment and telemedicine-based patient monitoring into cessation programs may enhance treatment effectiveness and support sustained abstinence in diverse clinical populations.

### Strengths and limitations

Key strengths of this study include its prospective design, relatively large sample size, and the use of standardized and widely validated psychometric instruments to assess depression and anxiety. The real-world clinical setting enhances external validity and closely reflects routine smoking cessation practice, in which pharmacological treatment is typically combined with structured behavioral support. Importantly, patient follow-up was reinforced through scheduled telemedicine-based contacts at two-week intervals, allowing continuous monitoring, timely counseling, and early identification of psychological distress or treatment-related issues. This hybrid follow-up model, integrating face-to-face care with telemedicine, likely contributed to improved treatment adherence and sustained engagement during the high-risk early phase of smoking cessation.

Several limitations of this study should be acknowledged. First, the observational real-world design precludes causal inference regarding the independent effects of smoking cessation, pharmacological treatment, and telemedicine-based follow-up on psychological outcomes. Treatment modalities were delivered according to routine clinical practice, and combination therapies were frequently used, limiting the ability to disentangle the specific contribution of individual pharmacological agents, including cytisine. Second, although validated self-report instruments were employed to assess depressive and anxiety symptoms, these measures do not substitute for structured psychiatric interviews and may be influenced by reporting bias, moreover as smoking cessation was not verified through exhaled CO, there remains a possibility of misclassification. Third, the absence of a non-telemedicine control group restricts conclusions regarding the incremental psychological benefits attributable specifically to structured telemedicine follow-up. Fourth, follow-up was limited to six months; therefore, long-term psychological trajectories and their relationship with sustained abstinence could not be evaluated. Finally, as the study was conducted in a single specialized smoking cessation clinic, generalizability to other healthcare settings and populations may be limited. Despite these constraints, the real-world prospective design and systematic longitudinal assessment of psychological outcomes represent important strengths and provide clinically relevant insights into contemporary smoking cessation care.

## CONCLUSIONS

This prospective real-world study demonstrates that smoking cessation care integrating pharmacological treatment, behavioral counseling, and structured telemedicine-based follow-up may be associated with meaningful improvements in depressive and anxiety symptoms during the early cessation period. These findings reinforce growing evidence that smoking cessation is linked to psychological benefit and underscore the importance of addressing mental health concerns as an integral component of tobacco dependence treatment. From a policy and service-delivery perspective, the results provide further evidence that supports the incorporation of routine mental health assessment and telemedicine-based monitoring into standard smoking cessation programs, in line with contemporary global recommendations. Such integrated care models may enhance treatment adherence, facilitate early identification of psychological distress, and support sustained abstinence, particularly in resource-constrained settings where scalable and cost-effective interventions are needed. Future research should focus on long-term psychological outcomes, comparative effectiveness of specific treatment components, and implementation strategies to optimize integrated cessation services across diverse healthcare systems.

## Data Availability

The data supporting this research are available from the authors on reasonable request.
